# Loading of erythropoietin on biphasic calcium phosphate bioceramics promotes osteogenesis and angiogenesis by regulating EphB4/EphrinB2 molecules

**DOI:** 10.1007/s10856-022-06644-9

**Published:** 2022-01-24

**Authors:** Yu Wang, Peng Wang, Qionghui Wu, Zhifan Qin, Zichao Xiang, Yuxian Chu, Jihua Li

**Affiliations:** State Key Laboratory of Oral Diseases & National Clinical Research Center for Oral Diseases & Dept. of Orthognathic & TMJ Surgery, West China Hospital of Stomatology, Sichuan University, Chengdu, 610041 China

## Abstract

Improving osteogenesis and angiogenesis using different cells and drugs is critical in the field of bone tissue engineering. Recent research has found that erythropoietin (EPO) plays an important role in both osteogenesis and angiogenesis. In this study, we grafted polydopamine and EPO onto the surface of biphasic calcium phosphate. The characterization and release property of the modified bioceramics were assessed. Cell proliferation, expression of osteoblastic and endothelial markers, and EphB4/EphrinB2 molecules were investigated while employing co-cultures of two different cells [rat vein endothelial cells (VECs) and rat bone marrow mesenchymal stromal cells (BMSCs)]. The modified bioceramics were finally implanted into the SD rats’ femurs and followed by investigating the bone defect repair efficacy and the expression of EphB4/EphrinB2 molecules in vivo. The results indicated that the modified bioceramics could control the release of EPO continuously. The osteogenesis and angiogenesis were improved along with the increased expression of EphB4/EphrinB2 molecules. The expression of EphB4/EphrinB2 molecules was also significantly increased in vivo and the bone defect was repaired effectively. Overall, our findings demonstrated that EPO loading on biphasic calcium phosphate bioceramics could promote both osteogenesis and angiogenesis. The results suggest that EphB4/EphrinB2 may be crucial in the process.

**Figure:**
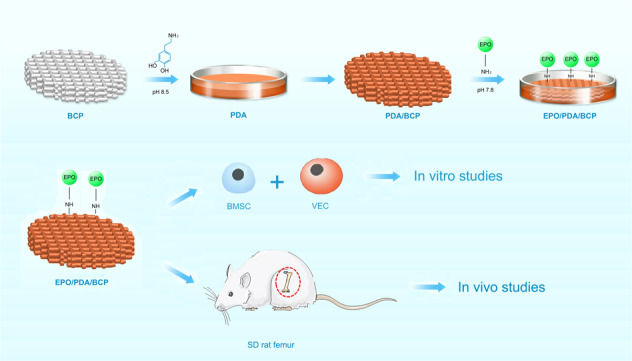
Graphical abstract

Graphical abstract

## Introduction

Calcium phosphate ceramics have been used as effective bone substitutes for the repair of surgical or traumatic defects. Biphasic calcium phosphate (BCP) consisting of two individual phases of calcium phosphate β-tricalcium phosphate (TCP) and hydroxyapatite (HA) in variable proportions is bioactive and similar with the natural bone in inorganic composition [1, 2]. Recently, a growing number of studies have suggested that surface modification of bioceramics with various proteins may play a key role. Bioceramic surface compositions can influence cell functions including cell attachment, differentiation, and proliferation [3–5]. Different methods of surface modifications including physical, chemical and treatments have been used to improve bioceramics’ surface physicochemical properties and cellular bioactivity [6–8]. Typical proteins like BMP-7 and bone morphogenetic protein-2 have been widely investigated for improving the bone formation of bioceramics [4, 5, 9, 10].

Erythropoietin (EPO) is a glycoprotein hormone with a molecular weight of 30,400 that controls red cell production and has been used clinically for the treatment of a variety of diseases such as chronic renal failure and anaemia [11]. Studies have also proved the effect of EPO on the coordinated regulation of osteogenesis and angiogenesis [12–14]. EPO can be applied locally as well as systematically to promote bone development in rabbit and rat models with critical-size defects [15, 16]. However, the cellular and molecular mechanisms by which EPO promotes osteogenesis, are still unclear. The EphB4/EphrinB2, JAK/STAT, PI3K, and mTOR signaling pathways may be possible factors in EPO-mediated osteogenesis [17–19].

EphB4, a member of the largest tyrosine kinases receptor EPO family, is expressed in osteoblasts and regulates osteogenesis with its cognate ligand EphrinB2 [20]. According to the proposed mechanism, EphB4 has a positive impact on osteoblasts, while EphrinB2 has a negative effect on osteoclasts and inhibits their activity [21]. Li et al. found that EphrinB2 interacted with the EphB4 receptor when EPO promoted the osteogenesis on ST2 cells and bone marrow stromal cells (BMSCs) [17]. Thus, we investigated the EphB4/EphrinB2 molecules in the EPO-induced osteogenesis in our study.

The administration of EPO on bioceramics has so far received little attention. Li et al. developed a composite scaffold containing HA and EPO to enhance osteogenesis and angiogenesis by upregulating the HIF-1/VEGF pathway [22]. However, the controlled release pattern of EPO was not enhanced in the composite scaffold. To achieve the long-term controlled release of EPO loaded on BCP ceramics, we employed a simple surface modification method based on polydopamine (pDA) formed by dopamine self-polymerization. Dopamine is a well-known neurotransmitter that forms pDA easily in a weak alkaline environment. pDA coating, which involves simply immersing materials in a dopamine aqueous solution, has shown promise in surface modification and protein surface immobilization [23, 24]. pDA coating is material-independent and can be applicable to nearly all kinds of substrates [25]. The amines could theoretically immobilize EPO onto pDA surfaces through Schiff base reactions. Furthermore, pDA can also help human mesenchymal stem cells differentiate into osteogenic cells by increasing the levels of osteogenic gene expression [26]. To speed up the osteogenic differentiation of MSCs, the biochemical environment of the bone extracellular matrix could be mimicked to increase calcium mineralization [27, 28]. Moreover, pDA is relatively stable and durable, especially under aqueous conditions [29].

Until now, no study focused on the direct loading of EPO on biomaterials via mussel-inspired coatings like pDA. In this study, we used sequential incubation of BCP bioceramics with pDA and EPO in an aqueous solution. The properties of the modified bioceramics were investigated by X-ray diffraction (XRD) and X-ray photoelectron spectroscopy (XPS). The cell morphology, proliferation, and adhesion as well as osteogenesis and angiogenesis activity were also evaluated both in vitro and in vivo.

## Materials and methods

### Preparation of BCP bioceramics

All reagents used were of analytical grade, purchased from Merck, India, and used without purification. Pure Ca(OH)_2_ and (NH_4_)_2_HPO_4_ were used as starting materials to synthesize HA powders. Ca(OH)_2_ (3.71 g) was added to 100 mL of deionized water and stirred for 5 h to produce a uniform suspension. Meanwhile, 2 mL (NH_4_)_2_HPO_4_ solution (0.3 M) was diluted with 8 mL of deionized water. The ratio of Ca/P was kept at 1.67 as HA. The diluted (NH_4_)_2_HPO_4_ solution was added drop-by-drop to the Ca(OH)_2_ suspension with stirring; a white HA gel formed at the end of the titration. The HA gel (20 mL) was diluted with 80 mL deionized water to form a suspension, and NH_4_OH was added until the pH of the suspension was 11. Finally, the suspension was treated hydrothermally at 150 °C for 3 h. The HA powder was then collected by centrifugation, washed with deionized water several times, vacuum filtered and dried at 60 °C for 24 h.

The synthesis of β-TCP powders started with mixing solutions of SMS-sodium metasilicate (Qualigens) and 0.6 M of Na_2_HPO_4_ in a 1:1 ratio. A 50 mM solution of ascorbic acid was added, the solution pH adjusted to 7.4 using a 10% acetic acid solution, and allowed to gel. After gelation, a 1 M CaCl_2_ solution was used as the supernatant for crystallization The experiments were conducted at 37 °C. After crystallization, the samples were harvested, washed with deionized water, and dried at 60 °C. These samples were hydrothermally treated at 150 °C for 3 h, collected by centrifugation, washed with deionized water, and dried at 60 °C for 24 h.

The BCP bioceramics were prepared with 30% HA and 70% β-TCP. The two types of CaP ceramic precursor powders previously prepared via hydrothermal method were mixed with an appropriate amount of 30 wt% H_2_O_2_, 40 wt% PVA, and 3 wt% MC solution followed by heating in a microwave oven at 100 °C for 2 h. The slurry was stirred well and then poured into a mold with a specific shape of water absorption. The slurry was then dried at 40 °C, and placed in a muffle furnace for 2 h at 1100 °C for obtaining a three-dimensional porous ceramic. The sintered ceramic pieces were cut into regular discs (2 mm in thickness, 12 mm in diameter) for in vitro studies and regular small cylinders (4 mm in thickness, 3.5 mm in diameter) for implantation in vivo. The porosity data of the materials was determined by mercury porosimetry recorded with a Micromeritics AutoPore III 9410 porosimeter (Micrometrics Instruments) and scanning electron microscopy (SEM) (Inspect F, FEI, The Netherlands) observation was also presented. The BCP bioceramics were divided into four groups based on the different subsequent treatments: BCP with pDA and EPO, BCP with pDA alone, BCP with EPO alone, BCP alone.

### pDA coating on bioceramics

The BCP bioceramics were immersed in dopamine HCl (Sigma-Aldrich, St. Louis, MO, USA) solution (2 mg/mL) at pH 8.5 (Tris-HCl buffer). The solution was vigorously shaken, and the polymerization reaction was carried out at room temperature for 24 h in the dark [30]. To remove the free dopamine monomers, the bioceramics were rinsed with double-distilled water.

### EPO immobilization on the pDA-coated bioceramics

The pDA-coated bioceramics were immersed in the solution of 100 U/ml EPO (Peprotech 100-64, USA) at 37 °C for 24 h to promote the conjugation reaction. The unimmobilized EPO was then rinsed with phosphate buffer saline (PBS) and double-distilled water.

### Surface characterization of different bioceramics

The XRD patterns of different bioceramics were determined using a glancing angle X-ray diffractometer with Cu-Kα radiation (Rigaku, Japan). The XRD was performed at 35 kV and 35 mA with a step size of 0.02° (2θ).

The composition of different bioceramics was investigated through XPS (PE PHI-5300, USA) experiments using focused monochromatized Al K radiation (hv = 1486.6 eV). A hemispherical analyzer was used for the analysis of the generated photoelectrons and the core level XPS spectra for O1s, S2p, N1s, and C1s was measured.

Fourier transform infrared spectroscopy (FTIR) (Nicolet 6700, Thermo Scientific, USA) from 4000 to 550 cm^−1^ using KBr pellets was also used to determine characteristic functional groups on the surfaces of different BCP ceramics.

### Controlled release of EPO

The double ligand enzyme-linked immunosorbent assay (ELISA) was carried out for investigating the EPO loading capacity on the surface of different bioceramics. Fifteen bioceramics of each group were immersed in 2 mL PBS buffer on a 100 r/min shaker. Then 1 μL solution of each group was collected and detected on 1, 4, 7, 10, and 13 days at three biologically relevant pH values between 6 and 8. After 1000-fold dilution, the concentration of EPO in the solution was measured using an EPO-ELISA kit (Cloud-Clone Corp. Wuhan, China). The standard solutions were used for plotting the standard curve. The procedure was carried out as directed by the kit instructions. Briefly, 100 μL standard, control, or sample solution was added to the relevant wells followed by their incubation at 37 °C for 1 h. Then, immunoreagent (100 μL) was added to each well and incubated at 37 °C for 1 h. The wells were washed three times and then 50 μL TMB stop solution was added. Finally, photometric absorption at 450 nm was used to determine the EPO concentration. The EPO amount was determined by comparing the control and sample absorbance to the standard curve.

### Cell culture on different BCP bioceramics

Primary BMSCs were isolated from the femurs of 4-week-old Sprague-Dawley rats (Animal Research Center, Sichuan University, China) and cultured in modified a-MEM medium (90%, Hyclone), fetal bovine serum (FBS, 10%) (FMGBio, Shanghai, China), and streptomycin (10 mg/mL, Hyclone) with 1% 10 kU/mL penicillin as described in our previous study [31]. Rat VECs were purchased from Procell Life Science&Technology Co., Ltd. (China) and were subjected to culturing in 90% modified a-MEM medium supplemented with 10% FBS. The cells were plated in a culture flask at a density of 5 × 10^4^ cells/mL and incubated at 37 °C with 5% CO_2_. The BMSCs from passage 3 and the VECs from passage 5 were co-cultured on different BCP ceramics, with 1 × 10^5^ cells and 2.5 × 10^5^ cells respectively.

### Cell morphology and proliferation on BCP bioceramics

Cells were stained with phalloidin and DAPI after 3 days of co-culturing of BMSCs and VECs, and cell actin elongation and cell distribution were studied while using fluorescence confocal microscopy (Olympus, Japan). For dead and living cell staining, Dulbecco’s Phosphate Buffered Saline (DPBS) solutions supplemented with calcein-AM (2 mL, 1 mg/mL) and propidium iodide (2 mL, 1 mg/mL) were used, respectively. After a 40 min incubation at 37 °C in 5% CO_2_, the samples were washed in DPBS and imaged using fluorescence confocal microscopy.

### Real-time PCR of EphB4/EphrinB2 molecules, angiogenesis-related and osteogenesis-related factors

We chose the rat β-actin gene as a reference and used primer 5.0 design primer software to design gene primers based on the corresponding gene mRNA sequence. The blast function in NCBI was used to detect the primers’ specificity at first. All primers used in this study were synthesized by TaKaRa biotechnology Co. Ltd. After co-culturing the BMSCs and VECs for 7 days, five samples were taken from each group to investigate the expression of osteogenesis-related genes, angiogenesis-related genes, and the EphB4/EphrinB2 molecules. A total RNA extraction kit (BioFlux, Tokyo, Japan) was used to extract total cellular RNA. To remove DNA contamination, the RNA was digested with gDNA Eraser, and complementary DNAs (cDNAs) were synthesized using the PrimeScriptTM RT reagent kit with gDNA Eraser (Takara, Japan). cDNA sample (1 μL) of each primer was taken out followed by gradient dilution (10–10^5^) with distilled water. The primers were then assessed for their amplification specificity using an ABI-7300 Real-time PCR system (Applied Biosystems, Foster, CA). Real-time PCR was conducted with SYBR^®^ Premix Ex Taq^TM^ II kit (Takara, Japan) using an ABI-7300 Real-time PCR system (Applied Biosystems, Foster, CA). The procedure for amplification was as: 95 °C for 30 s followed by 40 cycles for 5 s at 95 °C and 60 °C for 30–34 s. According to the manufacturer’s protocol, all procedures were carried out in a 20-μL reaction mixture. To ensure that there was no contamination, nuclease-free water was used as a negative control. The difference (△Ct) between the Ct of each target transcript and Ct of rat Actin was used to calculate relative quantitation. 2^−△△^Ct was used to measure the fold change in expression level.

### Western-blot analysis of EphB4/EphrinB2 molecules, angiogenesis-related and osteogenesis-related factors

Western-blot analysis of the co-cultured cells for 7 days was performed to evaluate the protein expression of EphB4/EphrinB2 molecules, angiogenesis-related and osteogenesis-related factors. On 10% SDS-PAGE gels, total proteins were isolated and then transferred to polyvinylidene fluoride blotting membranes. The membranes were incubated overnight with the following primary antibodies after blocking nonspecific binding with 5% BSA in Tween-Tris-buffered saline: ALP antibody (bs-6292R), CD31 antibody (bs-20321R), CD34 antibody (bs-0646R), Col-1 antibody (bs-10423R), EphB4 antibody (bs-6046R), EphrinB2 antibody (bs-10659R,), Runx2 antibody (bs-20003R), VEGF antibody (bs-0279R), and β-actin antibody (bs-0061R, 1:5000 dilution, Bioss Co. Beijing, China). β-actin antibody was used as an internal control for protein expression across samples. The incubation of the membranes was then carried out with horseradish peroxidase-labeled secondary antibodies for 2 h. ECL™ western-blot detection reagents were used to visualize the immunoreactive proteins on the blots. Detection of the signals was carried out with Image Station 4000 R (Kodak, Rochester, NY, USA) followed by their analysis with Image J (Rawak Software, Inc. Germany).

### Immunofluorescent staining of EphB4/EphrinB2 molecules

To verify whether EphB4/EphrinB2 molecules are essential in angiogenesis and osteogenesis of co-cultured cells mediated by EPO, Immunofluorescence analysis of EphB4/EphrinB2 was performed on the co-cultured cells for 7 days using the rabbit polyclonal to EphB4 antibody (bs-6046R, Bioss Co. Beijing, China) and rabbit polyclonal to EphrinB2 antibody (bs-10659R, Bioss Co. Beijing, China). Briefly, cells were fixed for 15 min with 4% formaldehyde, washed, and overnight incubated with primary antibodies. The cells were again washed and incubated with fluorescent secondary antibodies for 1 h. DAPI was used to incubate the cells for 5 min. Fluorescence confocal microscopy was used to observe the cells, and Image J was used to interpret the positive expression.

### Animals and surgical procedures

In this study, 3 months old forty adult SD rats (with a 280–300 g body weight) were used. The rats were regularly provided with food and kept on a non-peros schedule the night before the operation. A 10% chloral hydrate intraperitoneal injection at 3.0 mL/kg provided general anesthesia, while a 2% hydrochloric acid lidocaine injection provided local anesthesia. To reveal the femoral lateral epicondyle, a vertical incision was made above the bilateral knee joint. On the femoral lateral epicondyle, a cylindrical hole-like defect (depth 4 mm, diameter 3.5 mm) was drilled. The procedure was carefully carried out to avoid damaging the lateral epicondyle’s external structure.

The prepared BCP bioceramics were implanted into the defect. EPO/pDA/BCP, EPO/BCP, or pDA/BCP was implanted in the right leg and BCP in the left leg in three different groups of rats (*n* = 10). Another ten rats were used as a sham surgery party, with no bioceramics implanted. The periosteum and the outer tissue were then sutured and reset. To avoid infection, a 400,000 U/kg intramuscular injection of penicillin sodium was given 3 days after the procedure. Rats were sacrificed with an overdose of 10% chloral hydrate after 1 and 3 months for immune-histochemical examination and micro-computed tomography (CT) evaluation.

### Micro-computed tomography (CT) evaluation

A μCT 80 micro-CT system (Scanco Medical, Bassersdorf, Switzerland) was employed for scanning the femurs 1 and 3 months after surgery at 114 μA, 70 kV, and 700 ms integration time. Multi-level threshold procedures (700 for ceramics and 200 for bone) were used to distinguish femoral bone tissue followed by the reconstruction of the images with an anisotropic voxel size of 10 μm. The mean trabecular number (Tb.N), the ratio of bone tissue volume to total tissue volume (BV/TV), the mean trabecular spacing (Tb.Sp), and the mean trabecular thickness (Tb.Th) were determined using the image analyzer software of μCT 80.

### Histological analysis

One and three months after the operation, 5 rats of each group were sacrificed and the femurs were immersed in 4% neutral paraformaldehyde buffered solution for 2 days. Histological femoral tissue sections were acquired after decalcification, embedding and slicing. HE staining was conducted with Hematoxylin and Eosin Staining (HE) Kit (Beyotime, China) according to the user’s protocols. Histomorphometry was performed on transverse sections using Nikon ECLIPSE E600 stereomicroscope (Nikon, Tokyo, Japan), computer-coupled Nikon DXM1200 Digital Camera and NIS-Elements F2.20 image software.

### Immune-histochemical analysis

Five rats from each group were sacrificed 1 and 3 months after the operation, and their femurs were submerged in a 4% neutral paraformaldehyde buffered solution for 2 days. After decalcification, embedding, and slicing, histological femoral tissue sections were obtained. The sections of the decalcified tissue were subjected to immuno-histochemical staining. The antibodies used included anti-RUNX2 (ab76596), anti-VEGF (ab53465), anti-EphB4 (ab73259) and anti-EphrinB2 (ab140077) (Abcam). After 1 h of incubation with a peroxidase-labeled secondary antibody, the color reaction was developed using the diaminobenzidine method. To prevent nonspecific secondary antibody binding, negative control without primary antibody was used. Under a microscope (Leica DMI 6000B, Germany), the epitopes of the two genes was observed, and the positive rate was measured using Image J.

### Statistical analysis

All experiments were repeated at least five times unless stated otherwise. Graphpad Prism7 software (Graphpad Software, USA) was used to perform the statistical analysis, and the results were presented as mean ± SD. ANOVA *t*-test was used for comparison, and the statistically significant difference was set at *p* < 0.05.

## Results

### Surface characterization of bioceramics

**Fig. 1:**
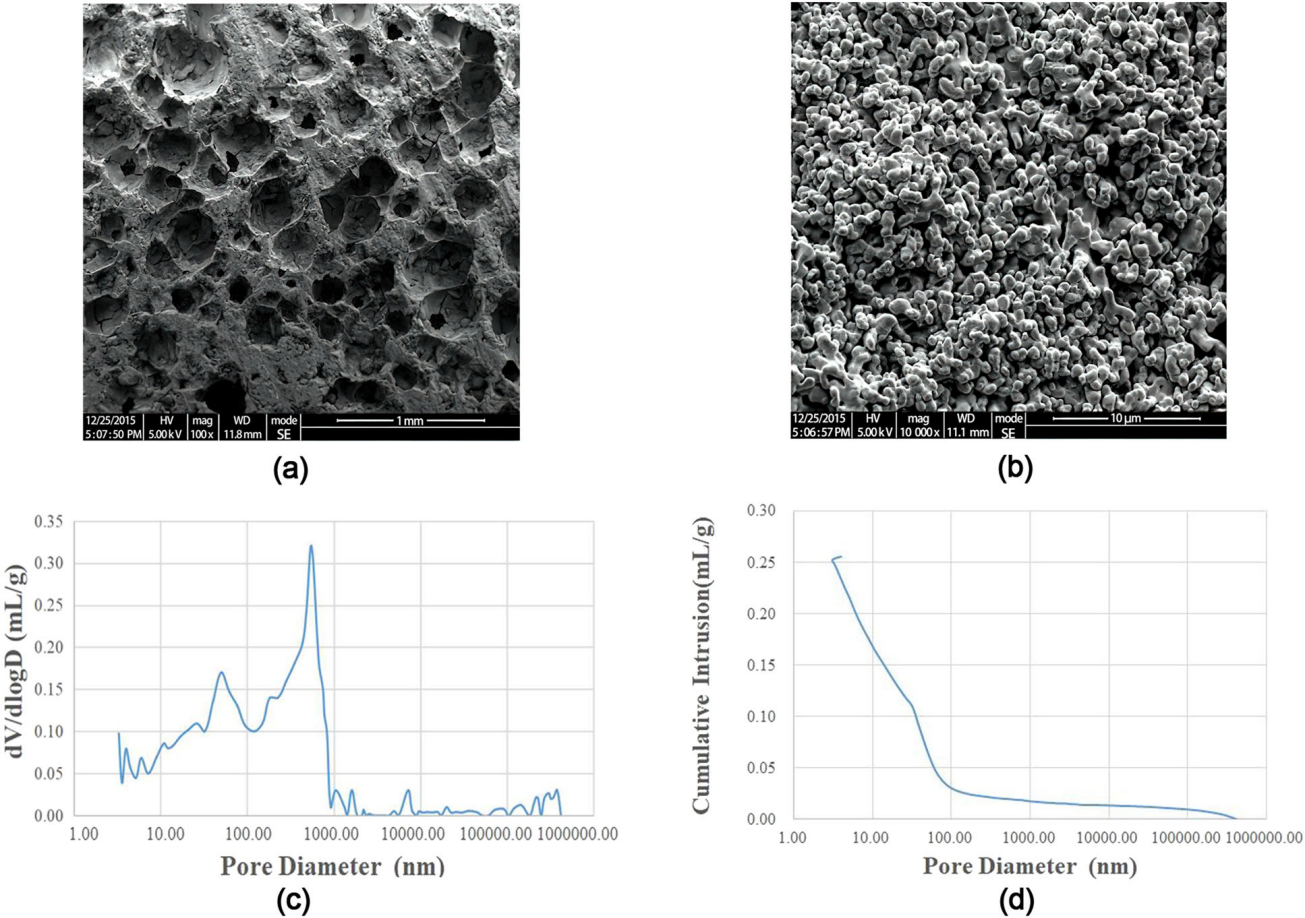
SEM images of BCP ceramics. The magnification was ×100 (**a**) and ×10,000 (**b**). Pore size distribution (**c**) and cumulative intrusion (**d**) of BCP obtained by mercury intrusion porosimetry

**Fig. 2:**
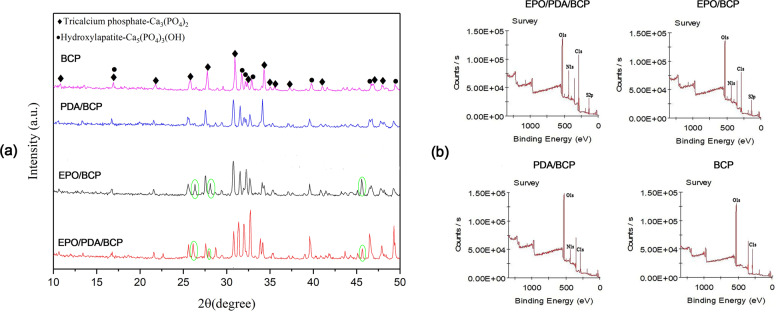
**a** XRD patterns of the surface-modified and unmodified BCP bioceramics. The circle indicates the additional diffraction peaks in three regions besides the characteristic peaks of BCP. **b** XPS spectra of the surface-modified and unmodified BCP bioceramics

Figure 1a, b show the SEM image of BCP. Figure 1c, d display the distribution of pore sizes and cumulative intrusion of BCP obtained by mercury intrusion porosimetry measurements. The BCP bioceramics had an average porosity of 63.6% and an average pore size of 95.7 nm. The two compositions of BCP, including HA and β-TCP, were identified in our study using XRD analysis. The crystal peak positions and sizes in the BCP and pDA/BCP bioceramics were identical. Aside from the characteristic peaks of BCP, the EPO/BCP bioceramics and EPO/pDA/BCP bioceramics had additional diffraction peaks in three regions. In addition, the peak sizes of characteristic peaks of BCP bioceramics also changed. The changes of the XRD curve preliminarily proved the successful grafting of EPO on BCP bioceramics (Fig. 2a).

Figure 2b shows the XPS spectra of the different bioceramics. In BCP bioceramics, only C1s and O1s peaks were observed, with no N1s or S2p peaks. The presence of an N1s peak in pDA/BCP bioceramics suggests the successful formation of pDA. The S2p peak only appeared on the surface of EPO/BCP and EPO/pDA/BCP bioceramics, suggesting that EPO was successfully grafted. The detailed element ratios are shown in Table 1. The EPO/BCP bioceramics had a nitrogen content of 12.77% on the surface, while the EPO/pDA/BCP bioceramics had a nitrogen content of 17.72%. The data further verified that EPO was successfully grafted on the pDA coating of the EPO/pDA/BCP bioceramics.

**Table 1:** Surface chemical composition of different BCP bioceramics

	C(%)	O(%)	N(%)	S(%)
EPO/PDA/BCP	35.44 ± 3.52	37.21 ± 5.10	17.72 ± 3.19	9.63 ± 2.63
EPO/BCP	29.91 ± 4.08	45.17 ± 5.39	12.77 ± 3.71	12.15 ± 2.44
PDA/BCP	19.84 ± 3.20	66.27 ± 6.72	13.89 ± 2.58	/
BCP	29.30 ± 4.16	70.70 ± 6.15	/	/

**Fig. 3:**
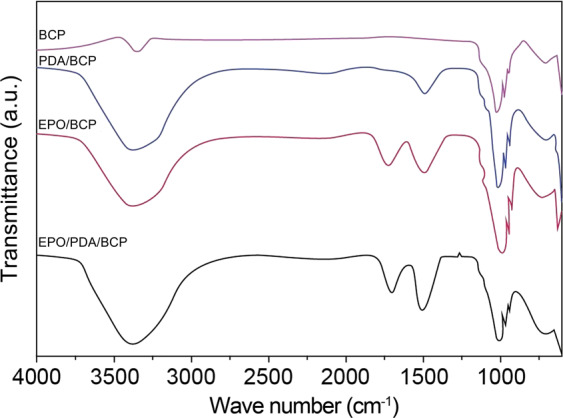
FTIR analysis of the surface-modified and unmodified BCP bioceramics

Primary functional group changes on the surfaces of different BCP ceramics were investigated by FTIR as shown in Fig. 3. Peaks were observed at 1019, 679–971, and 3357 cm^−1^ in BCP ceramics. FTIR spectra of pDA-treated BCP ceramics also contained peaks at 1533 and 3385 cm^−1^, corresponding to C–N bond stretching as well as N–H bond bending and N–H/O–H vibrations. These changes indicated successful polymerization of dopamine on the BCP ceramic surfaces. In FTIR spectra of EPO-treated BCP ceramics, in addition to peaks at 1533 (amide II band) and 3385 (N–H/O–H bonds) cm^−1^, peaks due to C=O stretching vibrations were observed at 1693 cm^−1^ (amide I band), which demonstrated the presence of EPO on the BCP ceramic surfaces (Fig. 3).

All of the above findings indicated that the PDA-coating remained attached to the surface of the bioceramic during biomolecule immobilization and that EPO was successfully attached to the surface after the treatment.

### Controlled release of EPO

**Fig. 4:**
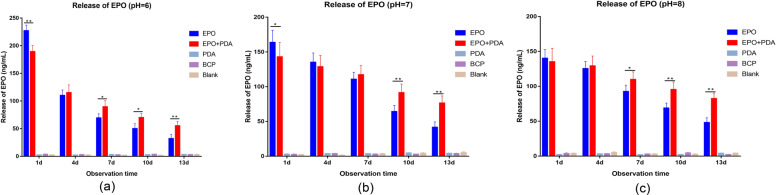
pDA-induced release of EPO on BCP bioceramics. The EPO/pDA was loaded and the released EPO concentrations at different time points and different pH (**a**: pH = 6; **b**: pH = 7; **c**: pH = 8) were quantified using ELISA. (**p < 0.01, *p < 0.05)

The release pattern of EPO at pH = 6, 7, and 8 from different bioceramics is shown in Fig. 4. The EPO release was slightly higher in acidic than those in neutral and basic environments on day 1, but lower on the other days. The EPO release at different biologically relevant pH values showed similar release pattern. When compared to the blank group, EPO release from BCP and pDA/BCP bioceramics was similar and low at different time points with no significant change. The EPO release from the EPO/pDA/BCP and EPO/BCP bioceramics decreased with time. The release rate from the EPO/BCP bioceramics was higher than the EPO/pDA/BCP bioceramics. On day 1, the EPO/pDA/BCP bioceramics released significantly less EPO than the EPO/BCP bioceramics (*p* < 0.05), but significantly more on days 10 and 13 (*p* < 0.01). The results of ELISA indicated the controlled release of EPO when conjugated with pDA.

### Cell morphology and proliferation on BCP bioceramics

**Fig. 5:**
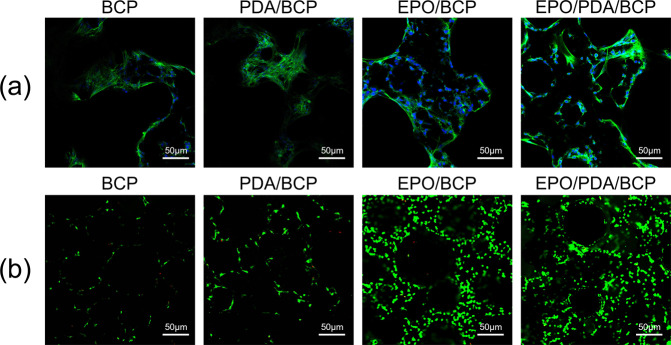
**a** Staining of co-cultured cells 3 days after inoculation: The blue color represents the DAPI-stained cell nucleus, while the green color represents the phalloidin-stained cytoskeleton of various BCP bioceramics. **b** Cell viability of co-cultured cells 3 days after inoculation on different BCP bioceramics using the live and dead assay. The dead and live cells are indicated by red and green colors, respectively

As shown in Fig. 5a, the cells adhered and gradually stretched on the surface of the bioceramics. Stretching out of the green cell actin filaments was observed, thus demonstrating the extended state of the cells on the surface. The cells spread more widely on the EPO/pDA/BCP and EPO/BCP bioceramics, showing better proliferation and cell viability. The results of live and dead cells are shown in Fig. 5b. On the surface of four bioceramics, a large number of live cells (stained in green) were found, with just a few dead cells (stained in red). The number of cells on the EPO/BCP and the EPO/pDA/BCP bioceramics was higher than that on the other two bioceramics. The number of cells of the EPO/pDA/BCP bioceramic was slightly higher than that of the EPO/BCP bioceramics.

### Real-time PCR of EphB4/EphrinB2 molecules, angiogenesis-related and osteogenesis-related factors

**Fig. 6:**
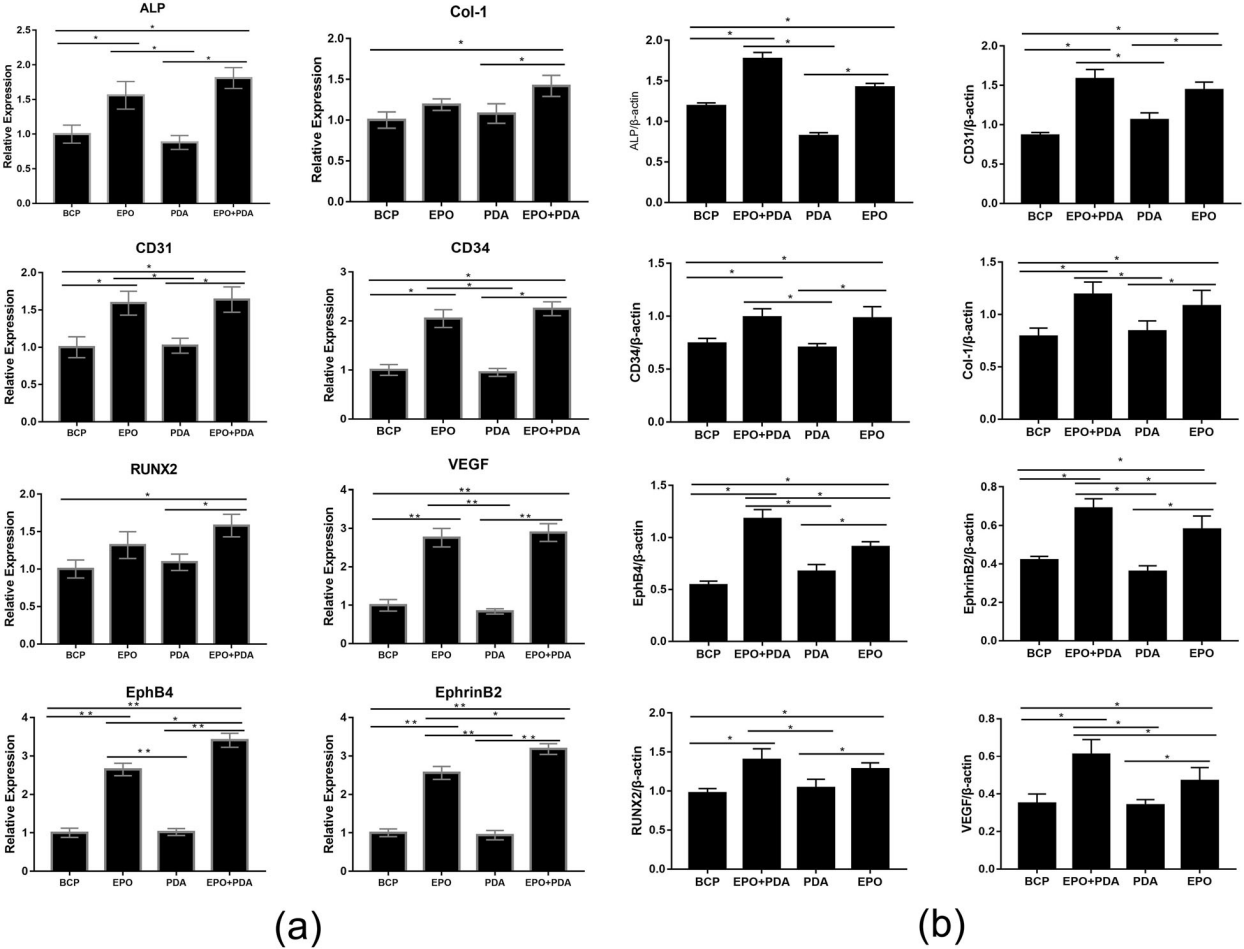
**a** The expression of osteogenesis and angiogenesis-related genes and EphB4/EphrinB2 signaling pathways of co-cultured cells on different BCP bioceramics on day 7. (**p < 0.01, *p < 0.05). **b** Western-blot experiments showed the expression of osteogenesis and angiogenesis-related proteins and EphB4/EphrinB2 signaling pathways proteins of co-cultured cells on different BCP bioceramics on day 7

There was no statistical difference in the expression of osteogenesis-related genes, angiogenesis-related genes, and EphB4/EphrinB2 related genes between the pDA/BCP group and the BCP group. When BMSCs and VECs were co-cultured for 7 days, the expression of all genes detected in the EPO/pDA/BCP group was significantly higher than that in the BCP group. The expression of all genes detected in the EPO/BCP group was significantly higher than that in the BCP group except for Col-1 and RUNX2. The EPO/pDA/BCP group had higher EphB4/EphrinB2 expression than the EPO/BCP group, but the expression of the other genes was not significantly different between the two groups. The results indicated that EPO promoted the expression of osteogenesis-related genes, angiogenesis-related genes, and EphB4/EphrinB2 related genes. Moreover, the application of pDA with EPO further promoted the expression of EphB4/EphrinB2 molecules (Fig. 6a).

### Western-blot analysis of EphB4/EphrinB2 molecules, angiogenesis-related and osteogenesis-related factors

The protein expressions were detected through the western-blot assay and the quantitative results are shown in Fig. 6b and Supplement.1. In general, higher protein expressions were observed in the EPO/BCP and EPO/pDA/BCP groups compared with the other two groups. The EPO/pDA/BCP group had higher protein expression of EphB4/EphrinB2 and VEGF than the EPO/BCP group. The expression profiles of genes and proteins showed a similar pattern.

### Immunofluorescent staining of EphB4/EphrinB2 molecules

**Fig. 7:**
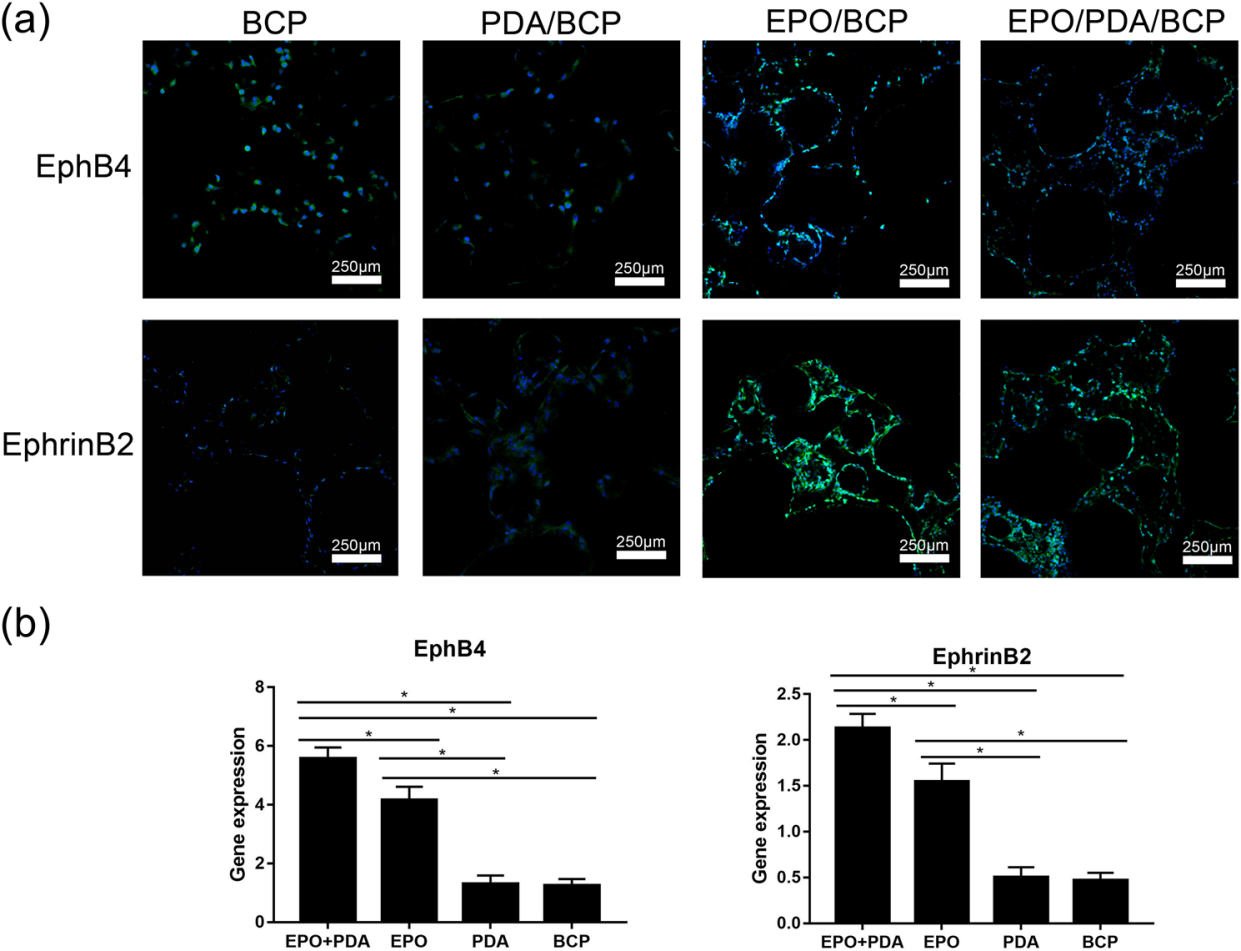
**a** Immunofluorescent staining showed the expression of EphB4/EphrinB2 signaling pathways of co-cultured cells on different BCP bioceramics on day 7. **b** The quantitative analysis of the immunofluorescent staining. (*p < 0.05)

Both the PDA/BCP and BCP groups had poor positive EphB4/EphrinB2 molecules expression, while the EPO/BCP and EPO/pDA/BCP groups had strong positive expression. A significant difference in expression was observed between the two groups, indicating that the EPO released by the EPO/pDA/BCP group could further stimulate the expression of the two molecules (Fig. 7).

### Micro-CT evaluation

**Fig. 8:**
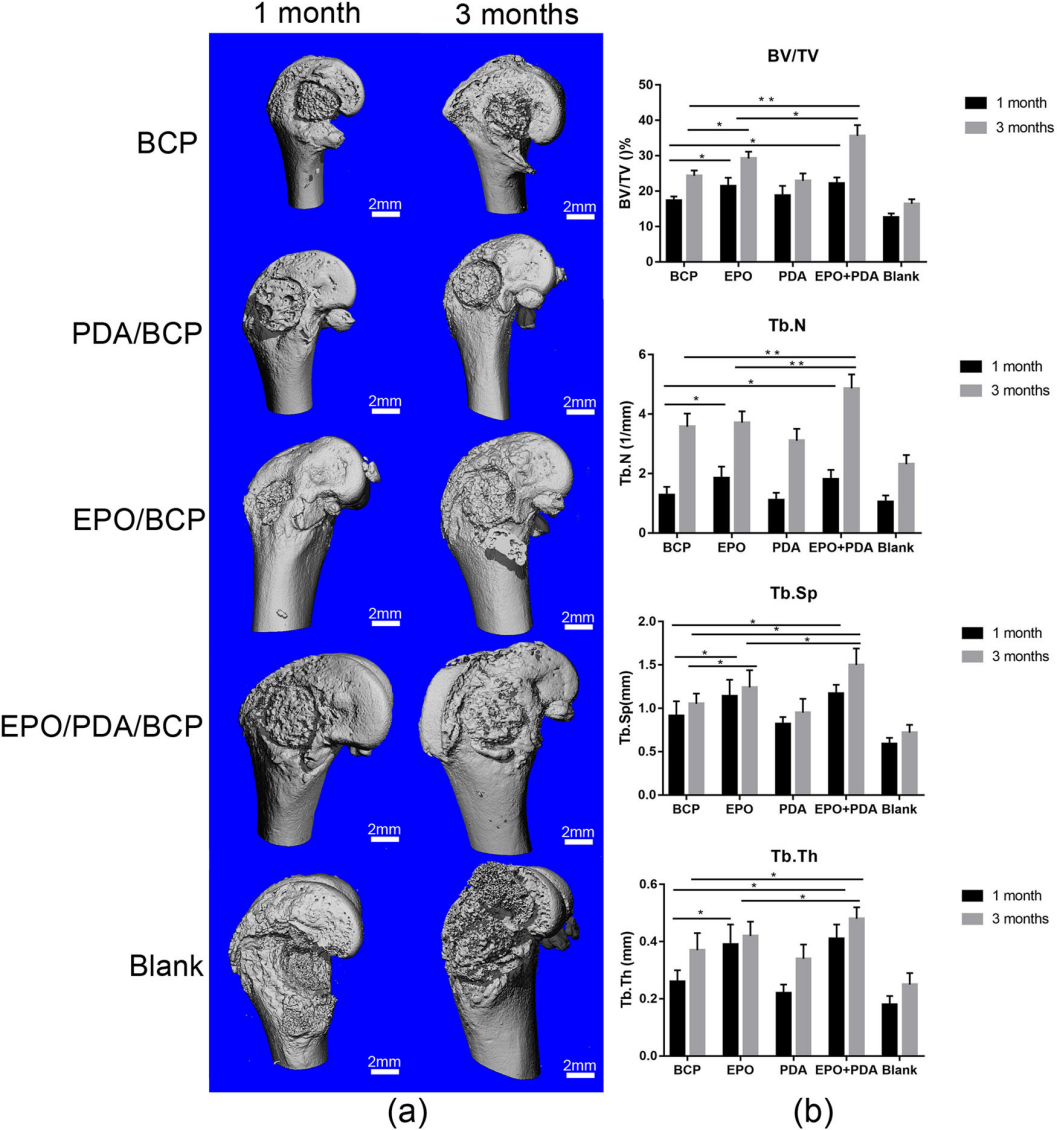
**a** Micro-CT images 1 and 3 months post operation. **b** Micro-CT evaluation 1 and 3 months post operation shows the mean trabecular number (Tb.N), the ratio of bone tissue volume to total tissue volume (BV/TV), the mean trabecular thickness (Tb.Th), and the mean trabecular spacing (Tb.Sp). (**p < 0.01, *p < 0.05)

One and months after surgery, the shape of the bioceramics in the rat femur was reconstructed three-dimensionally via the micro-CT evaluation (Fig. 8a). Multi-level threshold procedures of 200 and 700 were used as two thresholds to distinguish the bone and bioceramics. When compared to the sham surgery group, all of the bioceramics showed superior bone repair, and there was no difference between the pDA/BCP group and the BCP group. One month after surgery, the EPO/BCP group and EPO/pDA/BCP group had significantly higher bone mass and bone trabecula quality in the bone defect repair region than the BCP group. However, no statistical difference was observed between the EPO/BCP group and the EPO/pDA/BCP group 1-month post operation. Three months post operation, the bone mass as well as the bone trabecula quality of the EPO/pDA/BCP group were significantly higher than the EPO/BCP group and the BCP group. Only the bone mass, including the BV/TV and Tb.Sp, showed a statistically significant difference between the EPO/BCP and the BCP groups (Fig. 8b).

### Histological analysis

**Fig. 9:**
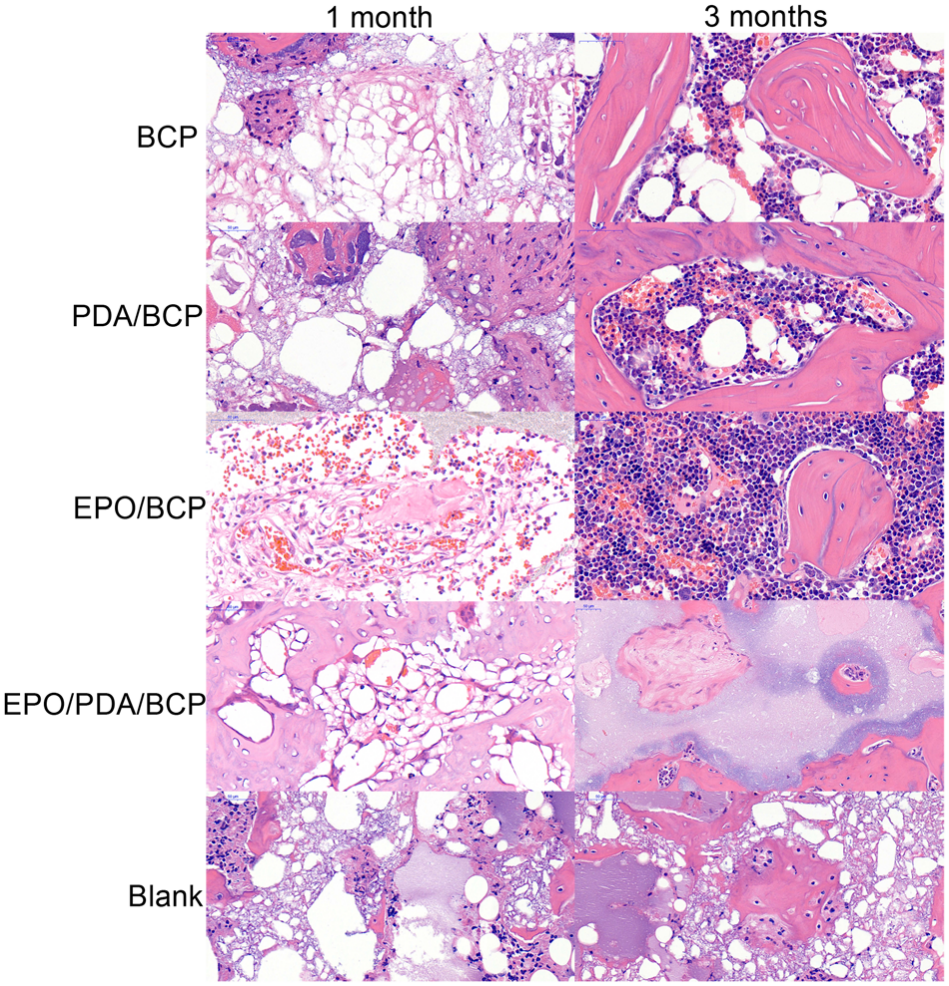
HE staining of rat femoral defect repair tissue sections 1 and 3 months post operation

Histological examination using HE staining (Fig. 9) showed the formation of fibrous and bone tissues at 1 and 3 months post operation. The evidence of new bone formation and the contact of new bone at the material interface were clearly observed. At 1-month post operation, a large number of fibers and a small amount of new-born bone were grown into the bone defect. More blood cells could be observed in the EPO/pDA/BCP and EPO/BCP groups than the other two groups. New bone formation increased with time. The newly formed bone at 3 months was larger in volume than that at 1 month after implantation. At 3 months post operation, a lot of new bone with a high degree of calcification as well as blood cells could be observed in the bone defect, especially in the EPO/pDA/BCP group.

### Immune-histochemical analysis

**Fig. 10:**
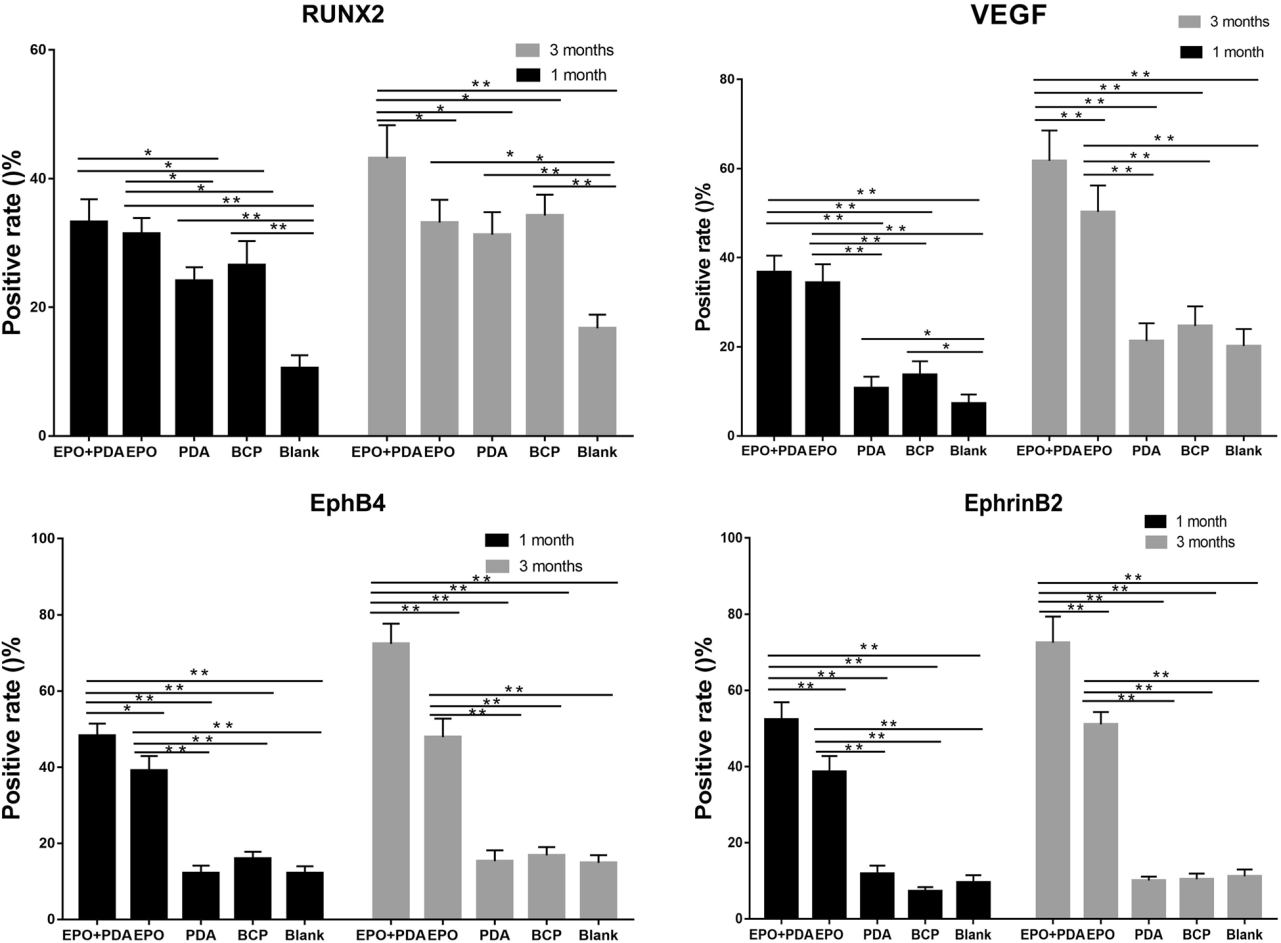
Quantitative analysis of immuno-histochemical staining of RUNX2, VEGF, EphB4, and EphrinB2 proteins 1 and 3 months post operation. (**p < 0.01, *p < 0.05)

The results of the quantitative immune-histochemical analysis are presented in Fig. 10. The positive rate of RUNX2 was higher in the EPO/pDA/BCP group and the EPO/BCP group at 1 month after the operation. At 3 months after the operation, the difference between the EPO/BCP group and the BCP group was not statistically significant. The EPO/BCP group, as well as the EPO/pDA/BCP group, had higher levels of VEGF expression 1 month after surgery. Three months after the activity, the EPO/pDA/BCP group had more strongly positive VEGF expression than the EPO/BCP group.

EphB4 and EphrinB2 were positively expressed in the EPO/BCP and EPO/pDA/BCP groups 1 month after surgery, however, no positive expression was observed in the other three groups. The EPO/pDA/BCP group had a more strongly positive expression of the two molecules 3 months after surgery than the EPO/BCP group.

## Discussion

The BCP scaffold composed of HA/β-TCP has been proven to be highly biocompatible and can promote the attachment, differentiation, and proliferation of osteoblasts [1, 32]. In a study by Tang et al., two different BCP with β-TCP to HA ratios of 30/70 and 70/30 were investigated and BCP with β-TCP to HA ratio of 70/30 showed earlier and significantly higher expression of osteogenesis-related genes [33]. At the histological stage, a higher proportion of β-TCP in BCP provided higher biocompatibility and osteoconductivity, so it was chosen in our experiment [34].

Surface characterization of BCP is the key factor for the determination of cell-material interactions. There have been many other studies using proteins to enhance the osteogenesis of BCP. Most researchers have used simple soaking to load the protein [4, 9, 10, 35]. Other materials such as aminobisphosphonates, pamidronate, and alendronate have also been employed for protein immobilization [5]. pDA may also act as a protein carrier, allowing the proteins’ release patterns to be regulated [36]. To achieve sustained release of EPO and promote osteogenesis and angiogenesis on the surface of bioceramics, we coated pDA on the surface of BCP bioceramics.

The bioceramics’ porosity and pore size were all examined. The modification with dopamine or EPO did not interfere with the material’s own structure. These findings were further confirmed by the XRD test. The positions of the peaks in the XRD curves of the four BCP bioceramics were basically similar. The XRD curve of pDA-treated BCP bioceramics did not change at all, indicating that the polymerization of dopamine on the surface of BCP ceramics did not affect its crystal structure. For the BCP ceramics treated with EPO, the XRD curves showed additional diffraction peaks at 2θ = 26.4°, 27.9°, and 45.8°. We speculated that these additional diffraction peaks might result from the unique protein crystal structure of EPO. The above changes initially demonstrated that EPO could be successfully grafted onto the surface of BCP ceramics. In order to further authenticate dopamine successful polymerization and the grafting of EPO, we used XPS to detect the C, O, N, and S elements. Only carbon and oxygen could be detected on the BCP ceramics without modification. Nitrogen appeared on the surface of the other three BCP ceramics due to the presence of dopamine or EPO. Due to the presence of disulfide bonds in the protein structure of EPO [11], sulfur was detected on the surface of BCP ceramics grafted with EPO. Moreover, we also found that the nitrogen content on the surface of EPO/pDA/BCP bioceramics increased compared with that of pDA/BCP or EPO/BCP bioceramics. From an elemental standpoint, the XPS results confirmed the effective modification of BCP ceramics with EPO or pDA. Primary functional group changes were presented by FTIR. The peaks observed at 1019, 679–971, and 3357 cm^−1^ in BCP ceramics corresponded to the antisymmetric vibration of the P–O–P bond, the symmetrical vibration of the P–O–P bond, and O–H vibrations, respectively. The peaks at 1533 and 3385 cm^−1^, corresponding to C–N bond stretching as well as N–H bond bending and N–H/O–H vibrations and the peaks due to C=O stretching vibrations were observed at 1693 cm^−1^ (amide I band) demonstrated the presence of dopamine and EPO on the BCP ceramic surfaces. Correspondingly, the peak intensities of the amide II band at 1533 cm^−1^ of EPO/pDA/BCP ceramics significantly outpaced those of EPO/BCP and pDA/BCP ceramics. Additionally, weak peaks corresponding to C–O bonds were observed at 1296 cm^−1^ (amide III band) and demonstrated successful loading of EPO on the EPO/pDA/BCP ceramic surface.

The EPO release pattern at different pH from the EPO/BCP and EPO/pDA/BCP bioceramics was studied by ELISA test. The rate of early release from EPO/BCP bioceramics was increased, and the pattern of release was similar to burst release. In contrast, the EPO released from EPO/pDA/BCP bioceramics was slower but more stable. Although the EPO release at different biologically relevant pH values showed similar release pattern, the EPO release was higher in acidic than those in neutral and basic environments on day 1, but lower on the other days. A controlled-release in acidic environment may be more difficult to obtain. The release period of EPO was still relatively short and required in vivo confirmation. The HA/TCP ratio in the BCP bioceramics used was 30/70. Thus, we assumed that increased TCP contents contributed to more obvious degradation of the scaffold and further release of the attached EPO [37].

The interactions of co-cultured BMSCs with VECs are already well known [38–40]. BMSCs can support VECs organization into prevascular-like structures in vitro [38, 39]. VECs also secrete osteogenic factors that induce bone formation [40]. The number of cells and the morphology of the cytoskeleton were observed using phalloidin and DAPI staining, and the live and dead method was used to assess cell proliferation and apoptosis. The number of dead cells on the surface of various BCP bioceramics is low, suggesting the materials’ low cytotoxicity. Not only did the number of cells on the two BCP ceramics treated with EPO significantly increase, but the cytoskeleton morphology was also significantly better than the other two BCP bioceramics. Surprisingly, the number of cells on the pDA/BCP bioceramics significantly increased compared with untreated BCP bioceramics. A study by Wu et al. suggested that the addition of Tris-HCl solution during the polymerization of dopamine might reduce the pH of the ceramic surface, accelerating the dissolution and precipitation of Ca and P ions in BCP ceramics [26]. However, the direct effect of dopamine on osteogenesis is unknown, and further research into the basic function and mechanism of dopamine is needed.

EPO is functionally related to bone metabolism. Studies have shown that anemic and polycythemic patients are both at risk for bone disorders [41]. EPO was also proved to promote the differentiation of osteoblasts and osteoclasts in vitro [16]. In addition, EPO showed an accelerated effect on bone fracture healing [42, 43]. Literature suggested the presence of EPO receptors on the surface of BMSCs. A proper amount of endogenous EPO could promote the differentiation of BMSCs, thus proving that EPO plays an important role in regulating osteogenesis [44]. The in vivo bone regeneration was observed through the Micro-CT test. Similar to our previous research, the results showed that the BCP ceramics had a better osteogenesis effect than the blank group. Further, the bone regeneration capability of the EPO/pDA/BCP and EPO/BCP bioceramics was much better than that of BCP ceramics without EPO. The EPO/pDA/BCP ceramics had a superior osteogenic effect 3 months after surgery. This can be attributed to the controlled release of EPO by the pDA. The pDA could strongly bind the EPO, thus resulting in long-term bond tissue regeneration.

Multiple signaling pathways might be involved in the EPO-induced osteogenesis [15–19, 45]. Pradeep et al. identified that EphB4 worked as an EPO receptor and EPO could induce EphB4-mediated functional and biological effects [46]. The interaction between EphB4/EphrinB2 signaling pathways and angiogenesis as well as osteogensis is complicated. In the angiogenesis process, EphB4 expresses more in venous cells, while EphrinB2 defines the venous and arterial boundaries of the developing vasculature [47]. EphB4 and EphrinB2 expression was found in growth plate osteoclastso and steoblasts, promoting the development of bone cells and bone growth [48]. It has been proposed that EphB4/EphrinB2 signaling pathways between osteoblasts and osteoclasts coordinate osteoclast-mediated resorption with osteoblast-mediated bone formation. Osx, Dlx5, and Runx2 are osteogenic regulatory factors induced by EphB4. EphrinB2 prevents apoptosis in osteoblast and suppresses osteoclast differentiation via RANKL [21, 49]. EphB4/EphrinB2 signaling pathways can also induce other regulatory pathways that control bone homeostasis, such as Wnt/-catenin, IGF-1, and ERK1/2, however, the exact crosstalk has not been thoroughly investigated [21, 50, 51].

RUNX2 is a major osteoblast-specific transcription factor and plays an essential role in osteogenesis [52]. VEGF is not only a major angiogenesis-related cytokine but also promotes osteoblast differentiation and increases the mineralization of new bone [53]. Therefore, we used RUNX2 and VEGF to indicate the osteoenesis and angiogenesis proteins expression in vivo. The results showed high consistency with the RT-PCR and western-blot results in vitro. The EPO/pDA/BCP group had significantly higher levels of VEGF and EphB4/EphrinB2 protein expression than the other groups. We speculated that the released EPO might directly affect the EphB4/EphrinB2 molecules. The use of EPO has also been shown to significantly increase the expression of VEGF [54]. We analyzed that VEGF protein might also be a downstream sensitive target of EPO. To confirm the role of EphB4/EphrinB2 molecules in EPO-induced bone and vascular regeneration, further research is required.

## Conclusion

This study aimed to look into the effects of EPO on BCP ceramics. The EPO loading efficiency and release pattern were also investigated in this study. The findings of in vitro and in vivo studies revealed that the scaffold system’s architecture clearly enhanced osteogenesis, angiogenesis, and the expression of EphB4/EphrinB2 molecules. The bioceramics EPO/pDA/BCP were found to be useful in preclinical studies, however, further research in a clinical setting would be needed to assess the bioceramics’ efficacy. The components of the bioceramics also need to be further investigated for better in vivo osteogenesis and angiogenesis applications.

## Supplementary Information

Supplement
