# Supplementary figures and images for: Loading of erythropoietin on biphasic calcium phosphate bioceramics promotes osteogenesis and angiogenesis by regulating EphB4/EphrinB2 molecules
Source: J Mater Sci Mater Med. 2022 Jan 24;33(2):19. doi: 10.1007/s10856-022-06644-9 (PMC8786765; doi:10.1007/s10856-022-06644-9)

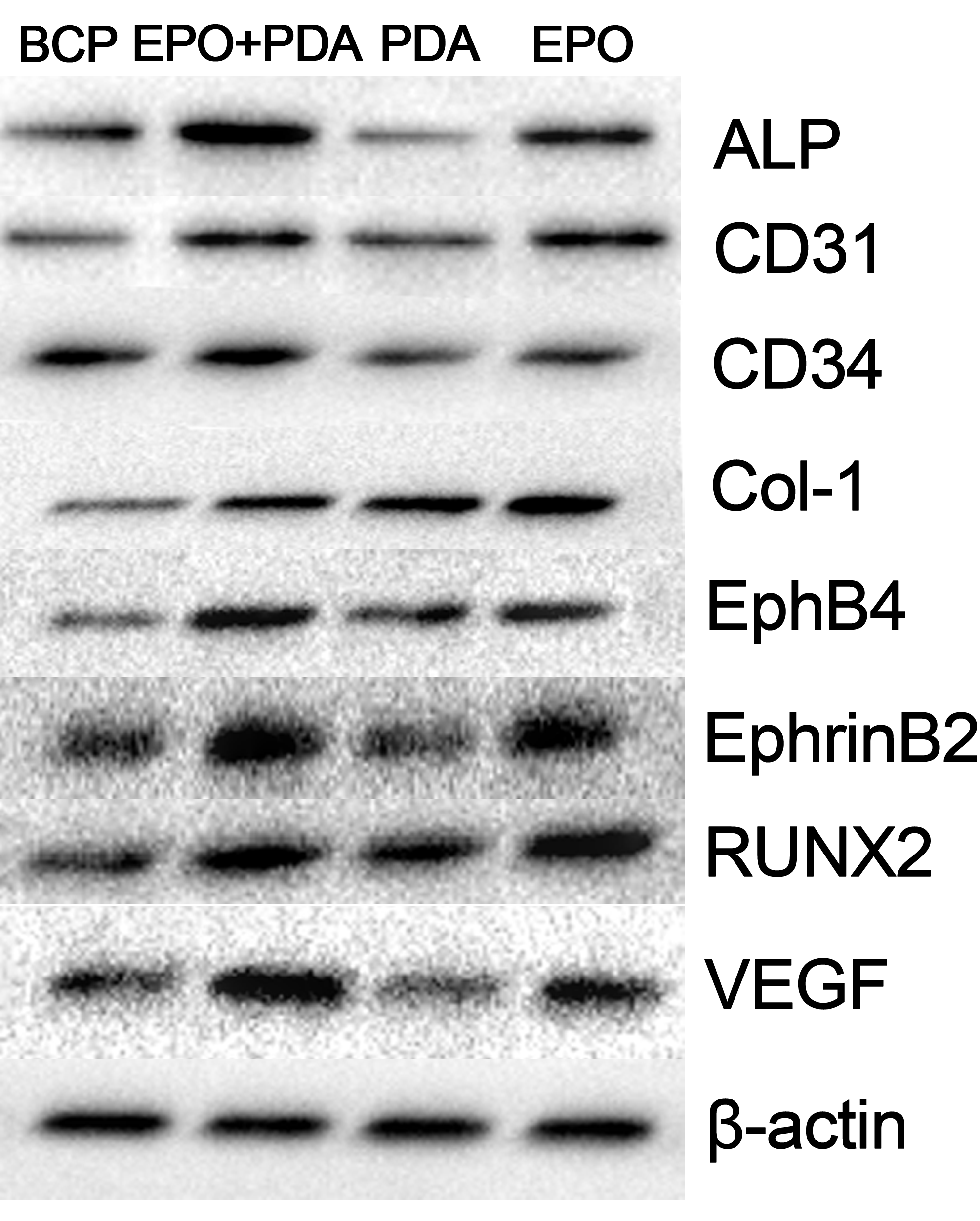

Supplement: Supplementary file 1 — Supplement [file 10856_2022_6644_MOESM1_ESM.tif]
